# Current infection prevention and control strategies of COVID-19 in hospitals

**DOI:** 10.3906/sag-2106-156

**Published:** 2021-12-17

**Authors:** Gamze KALIN ÜNÜVAR, Mehmet DOĞANAY, Emine ALP

**Affiliations:** 1 Department of Infectious Diseases and Clinical Microbiology, Faculty of Medicine, Erciyes University, Kayseri Turkey; 2 Department of Infectious Diseases and Clinical Microbiology, Faculty of Medicine, Lokman Hekim University, Ankara Turkey; 3 Scientific Committee Member, Ministry of Health, Ankara Turkey

**Keywords:** COVID-19, healthcare workers, infection, prevention and control

## Abstract

**Background/aim:**

Coronavirus disease 2019 (COVID-19), caused by the severe acute respiratory syndrome coronavirus-2 (SARS-CoV-2), has been appeared first in China since December 2019. Transmission of SARS-CoV-2 occurs primarily with droplets through coughing and sneezing and also occurs through inhalation of aerosolized secretions, which travel, remain suspended in the air longer.

**Materials and methods:**

Since early stages of the outbreak, COVID-19 cases have been described in healthcare workers (HCWs). However, in the early stages, the disease may be asymptomatic. This may lead to incorrect diagnosis or delayed diagnosis and may lead to the nosocomial spread of the virus. One of the most important causes of transmission among HCWs is being exposed to an aerosolized virus in a closed environment for a long time. It is possible to prevent and control the spread of COVID-19 in hospitals with outpatient treatment and triage.

**Results:**

Infection control measures, including wearing surgical masks, hand hygiene, and social distance are considered essential in preventing human-to-human transmissions of SARS-CoV-2. Immediate response and practices of infection control measures are critical for saving lives during an epidemic inside and outside the hospital.

**Conclusion:**

Analyzing current knowledge about the features of SARS-CoV-2 infection, screening, personal protection protocols, triage and psychological support practices for healthcare professionals can be promising in terms of controlling the infection.

## 1.Introduction

Coronavirus disease 2019 (COVID-19), caused by the severe acute respiratory syndrome coronavirus 2 (SARS-CoV-2), has been appeared first in China since December 2019 and then spread all over the world. World Health Organization (WHO) named the disease. “COVID-19” and declared it as a public health emergency in March 2020 [1,2]. As of June 2021, the global spread of the virus continues worldwide, with more than 173.331.478 confirmed cases and 3.735.571 deaths. SARS-CoV 2 is spread by inhalation of droplets World Health Organization. WHO Coronavirus (COVID-19) Dashboard https://covid19.who.int (Accessed on June 8, 2021). In addition, contact with virus-infected sources through eyes, nose, or mouth can also cause COVID-19 transmission. Airborne transmission is also possible after exposure to high concentrations of the aerosolized virus in a closed setting for a long time. After exposure, SARS-CoV-2 virus settles in the nose and throat, then can reproduce effectively. Hospitals are essential areas to provide medical care for patients and laboratory diagnoses. In hospitals, confirmed and suspected cases may be isolated to prevent viral transmission [3,4]. The transmission between patients and healthcare workers can occur in hospital settings during the SARS-CoV 2 outbreak. Some studies have reported that many the nosocomial outbreaks of COVID-19 are seen worldwide and led to difficulties in health systems. The nosocomial outbreak may cause significant morbidity and mortality among healthcare workers and patients. In the early stages of the disease, there may be few mild symptoms or may be asymptomatic. This may lead to incorrect diagnosis or delayed diagnosis and may lead to nosocomial spread of the disease. Studies have stated that detecting COVID-19 cases and preventing the spread may be difficult during the incubation period. Some patients without typical symptoms and fever may initially be admitted to different departments. This can create a risk to healthcare professionals [5,6]. The aerosolized virus in closed areas for a long time is one of the most important causes of transmission among HCWs. It is possible to control and prevent the spread of COVID-19 in healthcare facilities with outpatient treatment and triage. Initially, suspected cases can be identified and isolated by fever measurement, anamnesis, and evaluation of symptoms. Highly effective case isolation and contact tracing may control the outbreak of COVID-19 [7,8].

The COVID-19 infection prevention and control (IPC) guidelines have been advanced based on the experience of precautions and made interventions during MERS-CoV or SARS-CoV outbreaks. In addition, available literature has shown that SARS-CoV-2 is genetically similar to other SARS viruses. However, there are some differences from SARS-CoV other characteristics and terms of infectiousness. We reviewed information about the virus transmission, clinical presentations, and the transmissions while reviewing the guidelines. We collected different information about recommended control and prevention of COVID-19 Centers for Disease Control and Prevention. Interim Infection Prevention and Control Recommendations for Healthcare PersonnelDuring the Coronavirus Disease 2019 (COVID-19) Pandemic.https://www.cdc.gov/coronavirus/2019-nCoV/hcp/infection-control.html (Accessed on December 29, 2020), World Health Organization. Infection prevention and control during health care when novel coronavirus (nCoV) infection is suspected. January 25, 2020. https://www.who.int/publications-detail/infection-prevention-and-control-during-health-care-when-novel-coronavirus-(ncov)-infection-is-suspected-20200125 (Accessed on June 30, 2020) [9].

## 2. Transmission of SARS-CoV-2

Transmission of SARS-CoV-2 occurs primarily with droplets through coughing, sneezing, and talking and also through inhalation of aerosolized secretions, which travel, remain suspended in the air. The droplets may spread to about 4 m, so it may be the risk factor of infection to HCWs [10]. Aerosol-forming processes (such as intubation, sputum induction, and bronchoscopy) may increase the risk of transmission of the virus. Indirect transmission occurs when a susceptible person touches a contaminated surface and then touches his/her nose, eyes, and mouth. However, it is not thought to be a significant route of transmission [11,12]. Van Doremalen et al. showed that SARS-CoV-2 can survive on surfaces for a few days and in aerosols for 3 h [13]. Guo et al. also identified the SARS-CoV-2 virus on the shoe soles of HCWs [14]. This transmission may be important to spread the virus in healthcare settings. Patients should be screened for symptoms of COVID-19 upon entry into a health care setting. Suspected COVID-19 patients should be directed into a well-ventilated private room; such patients should not wait among other patients. Patients without symptoms should be questioned about any unprotected exposures to a person with COVID-19 within the last 14 days. If patients have had contact with suspected or confirmed COVID-19 cases, they may need to be quarantined. All of the guidelines and the studies to date recommend early diagnosis and isolation of patients to reduce exposures to SARS-CoV-2 [9,15]. 

## 3.Environmental disinfection and control

All guidelines recommend that environmental infection control procedures should be implemented to help reduce the spread of SARS-CoV-2. The routine cleaning and disinfection procedures of the CDC have been found appropriate for SARS-CoV-2 infections. Patients with suspected or confirmed COVID-19 should be placed in negative pressure rooms or a well-ventilated single-occupancy room with a closed door and dedicated bathroom; when this is not possible, patients with confirmed SARS-CoV-2 infection can be housed together [16].

Environmental services workers who are responsible for cleaning the areas should be trained for appropriate cleaning procedures. In order to reduce contamination, surface disinfection can be achieved by using a disinfectant such as 1/100 diluted bleach or chlorine tablet after cleaning with water and soap. It is recommended to be used for durable surfaces, as chlorine compounds can cause corrosion on surfaces. For sensitive surfaces, 70% alcohol can be used for surface disinfection by keeping it for 1 min. On the contaminated surfaces, disinfection is done with 1/10 diluted bleach or chlorine tablet and waiting until it dries Enfeksiyon Kontrolü ve İzolasyon. Hasta Odasına Giriş ve Hastaya Yaklaşım. Website; https://Covid19.Saglik.Gov.Tr/Eklenti/37699/0/Covid19rehberienfeksiyonkontroluveizolasyon. pdf. [17].

Hand hygiene and physical separation and are important in reducing the spreading of the virus in hospital settings. All guidelines recommend control measures such as barriers measures to manage patients, physical barriers in the reception area for triage, airflow management and closed systems for intubated patients. Air conditioners and fans should not be used in common areas, central ventilation systems should be used Airborne infection prevention and control technical briefs. Stop TB Partnership website. https://mailchi.mp/stoptb.org/infectious-diseaseprevention. (Published February 2020. Accessed on February 9, 2020), Prevention and control guidelines on novel corona virus pneumonia in Chinese. https://www.chinadaily.com.cn/pdf/2020/2.COVID19.Prevention.and.Control.Protocol.V6.pdf. [16,17].

## 4. Occupational risk for health care workers

In the database of WHO surveillance, HCWs definition includes physicians, nurses, health workers (physiotherapists, laboratory staff, patient transporters, etc.), and support staff, (laundry and cleaning personnel, reception clerks, etc.). HCWs are at high risk for COVID-19, and since early in the outbreak, COVID-19 cases in HCWs were reported. Before COVID-19 vaccines, according to the WHO data, 14% of COVID-19 cases were among HCWs Washingston State Department of Health. COVID-19 Confirmed Cases by Occupation andIndustry.https://www.doh.wa.gov/Portals/1/Documents/1600/coronavirus/covid_occupation_ industry_summary_2020-06-12.pdf (Accessed on July 14, 2020). Chou et al. reported that SARS-CoV-2 infection incidence in HCWs is between 0.4%–49.6%, and the prevalence of SARS-CoV-2 seropositivity ranged from 1.6%–31.6% [18]. In other reports, HCWs have accounted for 3.8%–19% of COVID-19 cases. Li et al. reported that there was no case among HCWs until January 1, 2020, and there was a 10% increase in 20 days after [19]. In a metaanalysis including the USA, China and European countries, it was reported that approximately 57.1% of healthcare workers were infected with COVID-19. In fact, 2736 healthcare worker deaths were documented with a death rate of 0–0.90/100.000 in the reporting countries until August 15, 2020 [20,21]. 

SARS-CoV-2 can spread from small liquid particles in the nose or mouth, ranging from droplets when the person coughs, sneezes, or talks. Also, the virus can be spreadable after inhalation or inoculation. In particular, inadequate use of PPE in infected patient rooms and exposure to infected workers in break rooms and common areas have been identified as major risk factors to HCWs [22]. 

The infection control precautions when caring for a patient with confirmed or suspected COVID-19 in the health care setting:

· When entering a patient’s room, a gown and gloves and PPE that provides respiratory, eye, and face protection should be worn by HCWs. 

· N95 and higher-level respiratory protection equipment are suggested rather than a medical mask to provide both barrier and respiratory protection.

A medical mask or a face shield can be used over the N95/FFP2 mask to reduce external contamination. It can be used for 8 h with transitions between patients without removing the mask. Masks can be hidden in a clean environment by wrapping them in a breathable paper bag or paper towel. It is not recommended to use a nylon bag for this purpose. It should not be used more than five times under these storage conditions. For example, the UK guideline recommends that PPE be used for about 2–6 h, while the ECDC guidelines recommend for up to 4–6 h Public Health England. Guidance on Infection Prevention and Control for COVID-19.https: //assets.publishing.service.gov.uk/government/uploads/system/uploads/attachment_data/file/990923/20210602_Infection_Prevention_and_Control_Guidance_for_maintaining_services) (Published on January 10, 2020), Infection prevention and control for the care of patients with 2019-nCoV in healthcare settings. ECDC technical report. https: //www.ecdc.europa.eu/sites/default/files/documents/nove-coronavirus-infection-prevention-controlpatients-healthcare-settings. (Published March 31, 2020. Accessed on April 27, 2020).

· For eyes or face protection, goggles or a face shield should be used because eyeglasses are insufficient.

· If it is not possible to follow up in an isolation room, it would be appropriate to cohort patients in a large room. However, the distance between patients and healthcare professionals may differ according to the guidelines. The protective distance recommendations are 1 m for WHO, 1.5 m for Australia, and 2 m for CDC World Health Organization. Mask use in the context of COVID-19. https://www.who.int/publications/i/item/advice-on-the-use-of-masks-in-the-community-during-home-care-and-in-healthcare-settings-in-the-context-of-the-novel-coronavirus-(2019-ncov)-outbreak (Accessed on December 14, 2020).

A systematic review suggests that the occupational risk may be increased in specific clinical settings by inadequate hand hygiene. A risk assessment for SARS-CoV-2 should be performed to determine the level of risk for SARS-CoV-2 occupational exposure, implement adequate measures to reduce it, and protect healthcare workers. The workplace risk levels may be helpful for occupational health services for potential occupational exposure to SARS-CoV-2. These classification of risk factors are shown in Table Infection prevention and control during health care when coronavirus disease (COVID-19) is suspected or confirmed. Interim guidance, 29 June 2020. Geneva: World Health Organization (https://www.who.int/publications/i/item/WHO-2019-nCoV-IPC2020. (Accessed on November 20, 2020).

**Table:** Risk levels, corresponding measures for prevention, and mitigation of occupational exposure to SARS-CoV-2 among health workers.

Lower-risk	Medium-risk	High-risk	Very high-risk
Without close contact and not to contact with people known or suspected of infection with SARS-CoV-2	Close contact with patients or visitors, not contacting people known or suspected of infection with SARS-CoV-2	Close contact with people who are known to be or suspected of infection with SARS-CoV-2 or contact with surfaces contaminated with the virus	With risk of exposure to aerosols of SARS-CoV-2, in settings where aerosol procedures are performed or working in crowded places without adequate ventilation, long working hours, inappropriate use of PPE for healthcare professionals

The precautions according to their level of risk have been recommended to prevent exposure of healthcare workers to SARS-CoV-2, following WHO guidelines. Because of medical conditions, older age, or pregnancy, some HCWs may be at risk for severe COVID-19 illness. They should not work with the medium, high, or very high-risk levels following WHO recommendations. In general, pregnant HCWs may continue to work in patient-facing roles until they give birth if all recommended personal protective equipment (PPE) is available. However, it is reasonable to limit exposure to patients with confirmed or suspected COVID-19, particularly during higher-risk procedures or unvaccinated. Some newly graduated or student HCWs may be at greater risk because they are unknown to control procedures or prevent errors while practicing. In particular, appropriate task delegation and role assignment can be considered for these healthcare professionals. Working for a long time without breaks and inadequate PPE leads to fatigue and inadequate compliance with infection prevention practices. According to WHO guidelines, adequate staffing levels and adequate infection prevention and control training are strongly recommended for effective IPC programs to prevent healthcare-associated infections [9,16].

The essential point of infection control measures to reduce transmission of SARS-CoV-2 includes early diagnosis, source control and isolation of suspected patients, environmental disinfection, and use of appropriate PPE. Generally, PPE is used for short periods when the exposure cannot be avoided or controlled otherwise. The research suggests that prolonged use of gloves and frequent hand hygiene may cause or aggravate hand eczema or latex allergy to sensitive people. For latex allergy, the use of non-latex or nitrile gloves is advised. Moisturizing creams help to decrease irritation [9,16,18]. 

Increasing inappropriate use of disinfectants in healthcare facilities can also cause toxic effects among healthcare workers and cleaners. HCWs involved in the application of disinfectants should be evaluated for medical contraindications, provided with adequate PPE. Spraying with disinfectants (such as in a tunnel, cabinet, or chamber) is not recommended under any circumstances [16].

In all health care facilities, the effectiveness of ventilation systems may be consulted to experts to assess and contain the spread of infected particles. Aerosol-generating procedures should be performed in rooms with appropriate air exchange capacities, and exposure to COVID19 without adequate protection should be investigated. HCWs should be reported if they exposure to COVID19 without adequate protection [9,22]. 

Vaccination is one of the strategies to reduce the risk of acquiring COVID-19 in health care settings, including vaccination of HCWs. Unless there is a contraindication such as allergic reactions, COVID-19 vaccine is indicated for all HCWs. The studies report that it reduces SARS-CoV-2 infections in HCWs. Some systemic signs and symptoms (fever, myalgia, headache, arthralgia, etc.) can occur after vaccination. It can be challenging to distinguish these manifestations from signs and symptoms of COVID-19. For this reason, it is essential to follow up after vaccination, and a COVID PCR test may be required for differential diagnosis. Screening of HCWs for symptoms and evidence of exposure for COVID 19 can be used in addition to the overall infection control program. It is essential to identify people with COVID-19 in healthcare institutions to reduce the transmission of the virus. A study reported that of 48 HCWs with confirmed COVID-19, 65% worked for approximately two days although they have the symptoms of COVID-19. This study determined that 17% of symptomatic healthcare workers were overlooked by more limited symptom screening (fever, cough, shortness of breath, sore throat, etc.) [23,24].

Whether HCWs have been vaccinated against COVID-19, where the exposure occurred, distance from the source patient, exposure time (>15 min in 24-h period), whether there is an aerosol-generating procedure, whether he uses personal protective equipment, whether the patient wears a mask should be questioned to assess exposure. Exposure is considered high risk if HCW was in the room during an aerosol-generating procedure, was not wearing all of the recommended PPE, and had prolonged close contact (within 2 m for ≥15 min over 24 h). All other exposures can be considered low risk. In HCWs, indications for quarantine depend primarily upon whether the exposure is considered high or low risk and whether the HCW had SARS-CoV-2 infection within the last 90 days [9,16].

## 5. Conclusion

It is impossible to be up-to-date for COVID-19 because new studies and guidelines are published daily. Infection control procedures should target all patient care procedures that address the risks of transmission by taking into account all possible routes of transmission. The main infection control measures, including wearing surgical masks, hand hygiene, and social distance are considered essential in preventing human-to-human transmissions of SARS-CoV-2. Immediate response and practices of infection control measures are critical for saving lives during an epidemic inside and outside the hospital. Analyzing current knowledge about the features of SARS-CoV-2 infection, screening, personal protection protocols, triage and psychological support practices for healthcare professionals can be promising in terms of controlling the infection.

## References

[ref1] Zhu N Zhang D Wang W Li X Yang B 2019 A Novel Coronavirus from Patients with Pneumonia in China The New England Journal of Medicine 382 727 733 10.1056/NEJMoa2001017 PMC7092803 31978945

[ref2] Zou L Ruan F Huang M Liang L Huang H 2020 SARS-CoV-2 Viral Load in Upper Respiratory Specimens of Infected Patients The New England Journal of Medicine 382 1177 1179 32074444 10.1056/NEJMc2001737 PMC7121626

[ref3] Wu Z McGoogan JM 2020 Characteristics of and Important Lessons from the Coronavirus Disease 2019 (COVID-19) Outbreak in China: Summary of a Report of 72 314 Cases from the Chinese Center for Disease Control and Prevention The Journal of The American medical Association 23 1239 1242 10.1001/jama.2020.2648 32091533

[ref4] Chou R Dana T Buckley DI Selph S Fu R Totten AM 2020 Epidemiology of and Risk Factors for Coronavirus Infection in Health Care Workers: A Living Rapid Review Annals of Internal Medicine 173 120 136 32369541 10.7326/M20-1632 PMC7240841

[ref5] Chou R Dana T Buckley DI Selph S Fu R Totten AM 2020 Update Alert: Epidemiology of and Risk Factors for Coronavirus Infection in Health Care Workers Annals of Internal Medicine 173 46 47 10.7326/L20-0768 PMC7304657 32515983

[ref6] Kluytmans-van den Bergh MFQ Buiting AGM Pas SD Bentvelsen RG 2020 Prevalence and Clinical Presentation of Health Care Workers With Symptoms of Coronavirus Disease 2019 in 2 Dutch Hospitals During an Early Phase of the Pandemic The Journal of the American Medical Association 3 209 209 10.1001/jamanetworkopen.2020.9673 PMC7243090 32437576

[ref7] Wong SCY Kwong RT Wu TC Chan JWM Chu MY 2020 Risk of nosocomial transmission of coronavirus disease 2019: an experience in a general ward setting in Hong Kong Journal Hospital Infection 105 119 127 10.1016/j.jhin.2020.03.036 PMC7128692 32259546

[ref8] Liu M Cheng SZ Xu KW Yang Y Zhu QT 2020 Use of personal protective equipment against coronavirus disease 2019 by healthcare professionals in Wuhan, China: cross sectional study British Medical Journal 10 2195 2195 10.1136/bmj.m2195 PMC7284314 32522737

[ref9] Lynch JB Davitkov P Anderson DJ Bhimraj A Cheng VC 2020 Infectious Diseases Society of America Guidelines on Infection Prevention for Health Care Personnel Caring for Patients with Suspected or Known COVID-19 10 10.1093/cid/ciaa1063 PMC7454357 32716496

[ref10] Guo ZD Wang ZY Zhang SF Li X Li L 2020 Aerosol and Surface Distribution of Severe Acute Respiratory Syndrome Coronavirus 2 in Hospital Wards Emerging Infectious Diseases Journal 26 1583 1591 10.3201/eid2607.200885 PMC7323510 32275497

[ref11] Çelebi G Pişkin N Çelik Bekleviç A Altunay Y Salcı Keleş A 2020 Specific risk factors for SARS-CoV-2 transmission among health care workers in a university hospital American Journal of Infection Control 48 1225 1230 32771498 10.1016/j.ajic.2020.07.039 PMC7409872

[ref12] Goldberg L Levinsky Y Marcus N Hoffer V Gafner M 2021 SARS-CoV-2 Infection Among Health Care Workers Despite the Use of Surgical Masks and Physical Distancing-the Role of Airborne Transmission Open Forum Infection Disease 8 36 36 10.1093/ofid/ofab036 PMC7928680 33732749

[ref13] Van Doremalen N Bushmaker T Morris DH Holbrook MG Gamble A 2020 Aerosol and Surface Stability of SARS-CoV-2 as Compared with SARS-CoV-1 The New England Journal of Medicine 382 1564 1567 32182409 10.1056/NEJMc2004973 PMC7121658

[ref14] Guo ZD Wang ZY Zhang SF Li X Li L 2020 Aerosol and Surface Distribution of Severe Acute Respiratory Syndrome Coronavirus 2 in Hospital Wards Emerging Infectious Disease 26 1583 1591 10.3201/eid2607.200885 PMC7323510 32275497

[ref15] Houghton C Meskell P Delaney H Smalle M Glenton C 2020 Barriers and facilitators to healthcare workers’ adherence with infection prevention and control (IPC) guidelines for respiratory infectious diseases: a rapid qualitative evidence synthesis Cochrane Database Systematic Reviews 4 3582 3582 10.1002/14651858.CD013582 PMC7173761 32315451

[ref16] Islam MS Rahman KM Sun Y Qureshi MO Abdi I 2020 Current knowledge of COVID-19 and infection prevention and control strategies in healthcare settings: A global analysis Infection Control and Hospital Epidemiology 41 1196 1206 32408911 10.1017/ice.2020.237 PMC7253768

[ref17] Ağalar C 2020 Protective measures for COVID-19 for healthcare providers and laboratory personnel Turkish Journal of Medical Sciences 50 578 584 32299205 10.3906/sag-2004-132 PMC7195977

[ref18] Chou EJ Schwartz NG Tobolowsky FA Zacks RLT Huntington-Frazier M 2020 Symptom Screening at Illness Onset of Health Care Personnel With SARS-CoV-2 Infection in King County The Journal of the American Medical Association 323 2087 2089 32301962 10.1001/jama.2020.6637 PMC7165316

[ref19] Li Q Guan X Wu P Wang X Zhou L 2020 Early Transmission Dynamics in Wuhan, China, of Novel Coronavirus-Infected Pneumonia The New England Journal of Medicine 382 1199 1207 31995857 10.1056/NEJMoa2001316 PMC7121484

[ref20] Gholami M Fawad I Shadan S Rowaiee R Ghanem H 2021 -19 and healthcare workers: A systematic review and meta-analysis International Journal of Infectious Diseases 104 335 346 33444754 10.1016/j.ijid.2021.01.013 PMC7798435

[ref21] Erdem H Lucey DR 2021 Healthcare worker infections and deaths due to COVID-19: A survey from 37 nations and a call for WHO to post national data on their website International Journal of Infectious Diseases 102 239 241 33130210 10.1016/j.ijid.2020.10.064 PMC7598357

[ref22] Chang D Xu H Rebaza A Sharma L 2020 Protecting health-care workers from subclinical coronavirus infection Lancet Respiratory Medicine 8 13 13 10.1016/S2213-2600(20)30066-7 PMC7128440 32061333

[ref23] Daniel W Nivet M Warner J Podolsky DK 2021 Early Evidence of the Effect of SARS-CoV-2 Vaccine at One Medical Center The New England Journal of Med 384 1962 1963 33755374 10.1056/NEJMc2102153 PMC8008752

[ref24] Benenson S Oster Y Cohen MJ Nir-Paz R. BNT162b2 2021 Vaccine Effectiveness among Health Care Workers. The New England Journal Med 384 1775 1777 33755373 10.1056/NEJMc2101951 PMC8008751

